# High-Fat Diet Induces Periodontitis in Mice through Lipopolysaccharides (LPS) Receptor Signaling: Protective Action of Estrogens

**DOI:** 10.1371/journal.pone.0048220

**Published:** 2012-11-02

**Authors:** Vincent Blasco-Baque, Matteo Serino, Jean-Noël Vergnes, Elodie Riant, Pascale Loubieres, Jean-François Arnal, Pierre Gourdy, Michel Sixou, Rémy Burcelin, Philippe Kemoun

**Affiliations:** 1 Institut National de la Santé et de la Recherche Médicale (INSERM), Toulouse, France; 2 Université Paul Sabatier, Unité Mixte de Recherche (UMR) 1048, Institut de Maladies Métaboliques et Cardiovasculaires (I2MC), Toulouse, France; 3 L.U. 51, Parodontites et Maladies Générales, Université Paul Sabatier, Faculté de Chirurgie Dentaire, 3, Chemin des Maraîchers, Toulouse, France; University of Tor Vergata, Italy

## Abstract

**Background:**

A fat-enriched diet favors the development of gram negative bacteria in the intestine which is linked to the occurrence of type 2 diabetes (T2D). Interestingly, some pathogenic gram negative bacteria are commonly associated with the development of periodontitis which, like T2D, is characterized by a chronic low-grade inflammation. Moreover, estrogens have been shown to regulate glucose homeostasis *via* an LPS receptor dependent immune-modulation. In this study, we evaluated whether diet-induced metabolic disease would favor the development of periodontitis in mice. In addition, the regulatory role of estrogens in this process was assessed.

**Methods:**

Four-week-old C57BL6/J WT and CD14 (part of the TLR-4 machinery for LPS-recognition) knock-out female mice were ovariectomised and subcutaneously implanted with pellets releasing either placebo or 17β-estradiol (E2). Mice were then fed with either a normal chow or a high-fat diet for four weeks. The development of diabetes was monitored by an intraperitoneal glucose-tolerance test and plasma insulin concentration while periodontitis was assessed by identification of pathogens, quantification of periodontal soft tissue inflammation and alveolar bone loss.

**Results:**

The fat-enriched diet increased the prevalence of periodontal pathogenic microbiota like *Fusobacterium nucleatum* and *Prevotella intermedia*, gingival inflammation and alveolar bone loss. E2 treatment prevented this effect and CD14 knock-out mice resisted high-fat diet-induced periodontal defects.

**Conclusions/Significance:**

Our data show that mice fed with a diabetogenic diet developed defects and microflora of tooth supporting-tissues typically associated with periodontitis. Moreover, our results suggest a causal link between the activation of the LPS pathway on innate immunity by periodontal microbiota and HFD-induced periodontitis, a pathophysiological mechanism that could be targeted by estrogens.

## Introduction

The prevalence of Type 2 diabetes (T2D) has dramatically increased over the past decade both in developed and developing countries. Furthermore, the complications of this metabolic disease are nowadays major causes of morbidity and mortality [1]. The pathophysiology of T2D is characterized by a low-grade chronic inflammation [2], with the release of inflammatory cytokines by innate immune cells, mainly macrophages and dendritic cells, that impair insulin action [3]. It was recently suggested that the intestinal microbiota contributes to the development of obesity and insulin-resistance [4], [5]. A switch from a normal diet towards a fat-enriched diet, where the daily amount of dietary fibers is reduced, was associated with a change in the ecology of the intestinal microbiota [6], [7] with an increase in gram-negative bacteria. The activation of the immune system by gram-negative bacteria depends on specific pattern recognition receptors (PRRs) such as Cluster of differentiation 14/Toll-like receptor-4 (CD14/TLR4) [8]. CD14/TLR4 knockout mice are protected against the metabolic impact of a high-fat diet (HFD) [4]. Hence, the interaction among gram-negative pathogens and the immune system is a key factor for the development of metabolic diseases [4], [9]. Interestingly, periodontitis, a chronic infection of the soft and hard tissues supporting the tooth, is caused by gram-negative capnophilic bacteria [10]. This disease is characterised by an inflammation and a loss of both soft and hard tissues of the periodontium (e.g. the periodontal tissues) that protect the roots of the tooth and anchor them to the jaws. Most pathogens involved in periodontitis have been identified, and many of them are also known to be involved in metabolic diseases [5] and many systemic diseases [11], [12]. *Prevotella intermedia* (Pi) is a key periodontal pathogen inducing innate immune responses partly involved in deep periodontal tissues destruction [13] The frequency of *Pi* detection in the periodontal pocket is higher in diabetic than in healthy subjects [14]. Another periopathogen, *Fusobacterium nucleatum* (Fn), was detected in human carotid endarteriectomy specimens and is thought to exert atherogenic effects [15]. Apolipoprotein E knockout mice (ApoE^−/−^) infected by *Fn* display increased lipid depots in the arterial wall compared with controls [16]. Indeed, *Fn* systemic infection increases plasma levels of total cholesterol and LDL [17]. Thus, it can be postulated that diet-induced metabolic diseases may favor the development of periodontitis.

We recently showed that estrogens are key players in the control of metabolic diseases involving immune regulation [18]. Furthermore, numerous epidemiological studies strongly suggest that estrogen deficiency is linked to the appearance of periodontal diseases (PD) [19]. As previously reported, a significant increase in the incidence of PD is associated with the menopause, which is considered as the main physiological cause of estrogen depletion [20], [21], [22]. Moreover, it has been suggested that hormonal replacement therapy can protect menopausal women against periodontitis [23]. However, the influence of these sexual steroid hormones on the occurrence of PD in a dysmetabolic and inflammatory context has not been addressed to date.

In this study, we investigated whether a high-fat diet, known to induce inflammation-mediated insulin-resistance and glucose-intolerance, as previously described [18], would promote the development of PD in ovariectomised mice, and whether estrogen administration would regulate this process. Our data reported herein demonstrate that HFD-induced metabolic disturbances were associated with the occurrence of periodontitis, and that chronic estrogen administration, as well as the deletion of CD14, strongly prevents the HFD-induced defects of periodontal tissue in mice.

## Materials and Methods

### Ethical Statement

All animal experimental procedures were approved by the local ethical committee of Rangueil University Hospital, INSERM BP 84225, 31432 Toulouse under the authorization number “C 31 555 07”, and the Ethical committee of Purpan Hospital (Toulouse).

**Figure 1:**
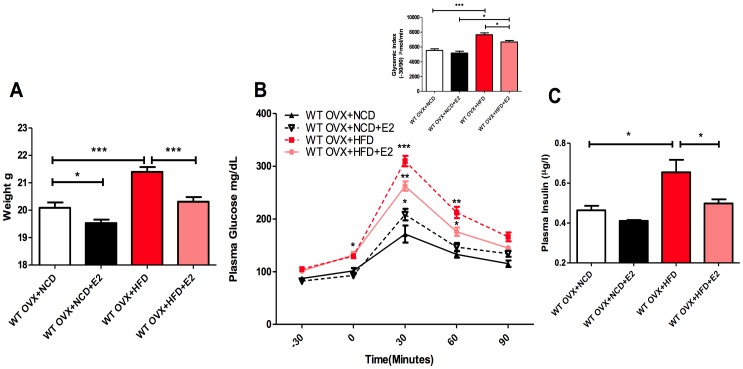
E2 administration prevents HFD-induced diabetic disorders. A ) Body weight was assessed in 8-wk-old mice 4 weeks of diet: WT OVX+NCD (n = 27), WT OVX+NCD+E2 (n = 16), WT OVX+HFD (n = 15) and WT OVX+HFD+E2 (n = 15). **B**) Time course of glycemia (mg/dl) during an IPGTT. The inset represents the glycemic index for each group. **C**) Fasted plasma insulin concentration (µg/l) after 4 weeks of diet: WT OVX+NCD (n = 6), WT OVX+NCD+E2 (n = 6), WT OVX+HFD (n = 6) and WT OVX+HFD+E2 (n = 6). *P<0.05, **P<0.01, ***P<0.001 (one-way ANOVA followed by Tukey test for **A** and **C**; and Two-Way ANOVA with Bonferroni’s post-test for **B**). Results are presented as means ± SEM.

### Animals and Experimental Procedures

C57BL6/J wild-type (WT) (Charles River, L’Arbres, France) and *CD14*-deficient (*CD14*KO) female mice born in our animal facilities were group-housed (five mice per cage) in a specific pathogen-free controlled environment (inverted 12-hr daylight cycle, light off at 10∶00 a.m.). Four week-old mice were ovariectomised (OVX) and then subcutaneously implanted with pellets releasing either placebo or 17β-estradiol (e.g. E2, 0.1 mg for 60 days, 80 µg/kg per day; Innovative Research of America, Sarasota, FL) [18] under general anesthesia. Mice have then been fed with a normal chow diet (i.e. NCD; energy content: 12% fat, 28% protein, and 60% carbohydrate; A04, Villemoisson sur Orge, France) or a diabetogenic, high-fat carbohydrate-free diet (i.e. HFD; energy content: 72% fat (corn oil and lard), 28% protein and less than 1% carbohydrate; SAFE, Augy, France) for four weeks. This particular diet has been developed to induce a diabetic state after 4 weeks, as previously described [9]. Animals were divided into eight groups: WT OVX+NCD with placebo (n = 27), WT OVX+NCD+E2 (n = 16), WT OVX+HFD with placebo (n = 25), WT OVX+HFD+E2 (n = 25), *CD14KO* OVX+NCD with placebo (n = 6), *CD14KO* OVX+NCD+E2 (n = 6), *CD14*KO OVX+HFD with placebo (n = 12) and *CD14*KO OVX+ HFD+E2 (n = 12). At the end of experiment, mice were sacrificed by cervical dislocation; tissues were collected and snap-frozen in liquid nitrogen.

**Table 1:** Positive bacterial cultures : intergroup comparisons among ovariectomised mice.

Bacterial cultures	Effect of diet			Effect of E2 supplementation				Effect of CD14 deletion		
	WT OVX NCD	WT OVX HFD	p^1^	WT OVXHFD		WT OVXHFD E2	p^1^	WT OVX HFD	*CD14*KO OVX HFD	p^1^
	n = 27 (%)	n = 25 (%)		n = 25 (%)	n = 25 (%)			n = 25 (%)	n = 12 (%)	
***Fusobacterium nucleatum*** ** (Fn)**	0 (0)	1 (4.0)	0.48	1 (4.0)	7 (28.0)		* **0.04**	1 (4.0)	0 (0)	1.00
***Prevotella intermedia*** ** (Pi)**	1 (3.7)	4 (16.0)	0.18	4 (16.0)	0 (0)		0.11	4 (16.0)	1 (8.3)	1.00
**Both Fn + Pi**	1 (3.7)	6 (24.0)	* **0.04**	6 (24.0)	0 (0)		** **0.02**	6 (24.0)	3 (25.0)	1.00
**Fn and/or Pi**	2 (7.4)	11 (44.0)	** **<0.01**	11 (44.0)	7 (28.0)		0.37	11 (44.0)	4 (33.3)	0.72

1 Fischer exact tests p-values, significant at p<0.05.

The table shows the qualitative analysis of periodontal microbiota in each group: WT OVX+NCD (n = 27), WT OVX+HFD (n = 25), WT OVX+HFD+E2 (n = 25) and *CD14*KO OVX+HFD (n = 12).

* P<0.05,

** P<0.01 (Fischer exact tests).

### Intraperitoneal Glucose-tolerance Test (IPGTT)

Six-hour–fasted mice were injected with glucose into the peritoneal cavity (1 g/kg) after 4 weeks of HFD diet. Blood glucose was measured with a glucometer (Roche Diagnostics, Meylan, France) on 3.5 µl of blood collected from the tip of the tail vein at −30, 0, 30, 60, and 90 min after the glucose injection [9].

**Figure 2:**
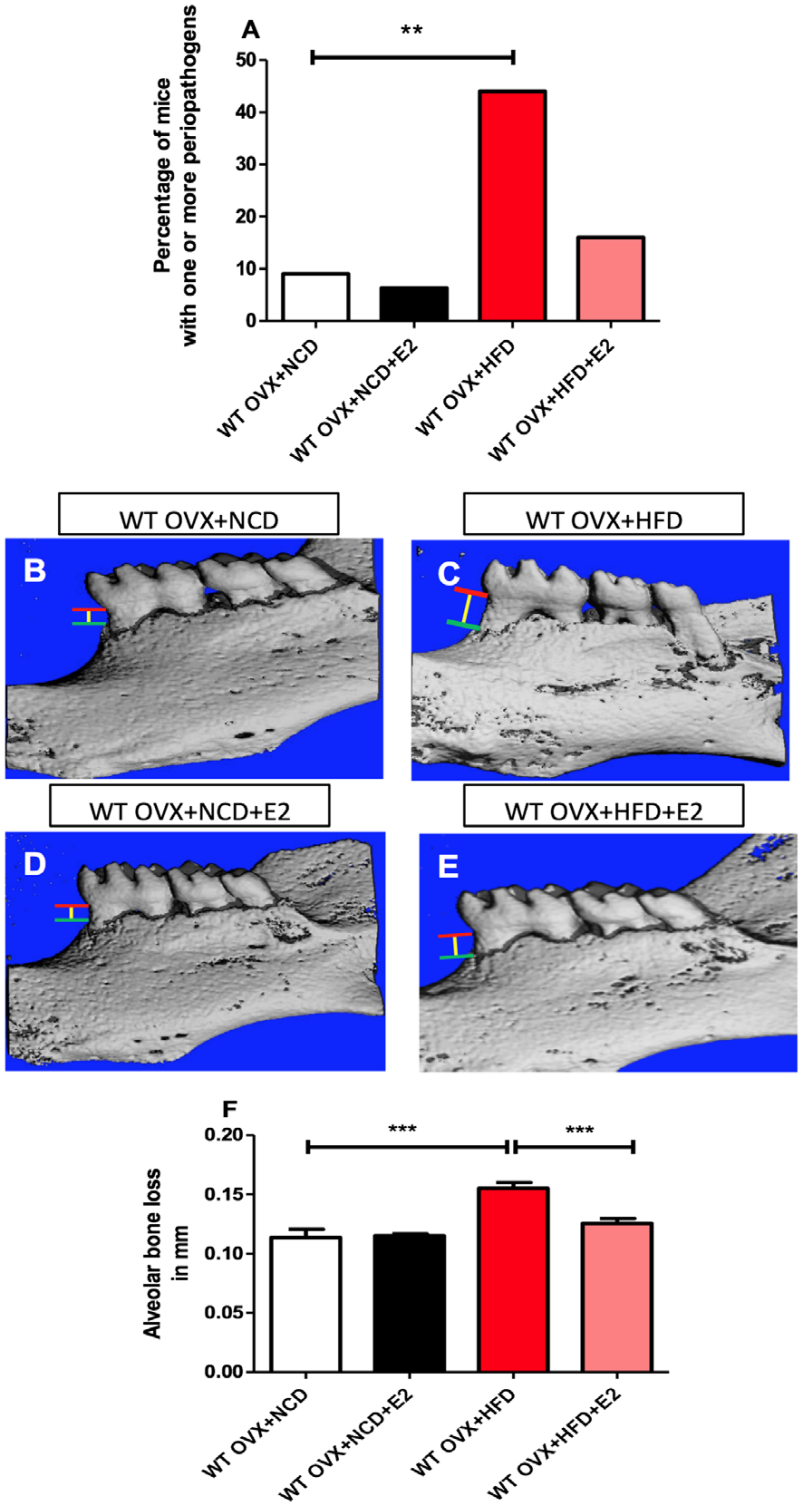
HFD induced periodontal disease in WT mice: reversal effect of E2-treatment. A) The occurrence of periodontal pathogens was analysed in 8-wk-old mice after 4 weeks of diet : WT OVX+NCD (n = 27), WT OVX+NCD+E2 (n = 16), WT OVX+HFD (n = 15) and WT OVX+HFD+E2 (n = 15). **B–G**) Hemi-mandible from each group, as reconstructed by the micro-CT. **F**) CEJ (red line: cemento-enamel junction)-ABC (green line: alveolar bone crest) distance to represent alveolar bone loss (yellow line) (n = 8 for each group). *P<0.05, **P<0.01, ***P<0.001 (one-way ANOVA followed by Tukey test). Results are presented as means ± SEM.

### Plasma Insulin Concentration

To assess plasma insulin concentration, 20 µl blood were sampled from the tip of the tail vein of 6-hr-fasted mice. The plasma was separated and frozen at −80°C. 5 µl plasma were used to determine the insulin concentration with an Elisa kit (Mercodia, Uppsala, Sweden) and following the manufacturer’s instructions.

**Figure 3:**
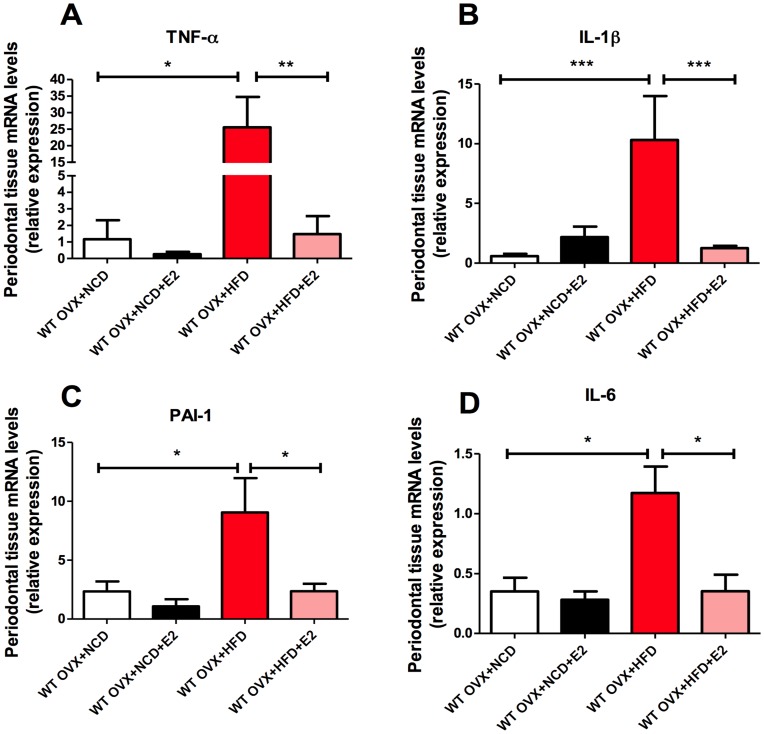
E2 decreases the expression of gingival inflammatory mediators in HFD-fed ovariectomised (OVX) mice. mRNA synthesis of TNF-α (**A**), IL-1β (**B**), PAI-1(**C**) and IL-6 (**D**) in gingival tissue. *P<0.05, **P<0.01, ***P<0.001 (one-way ANOVA followed by Tukey test). Results are presented as means ± SEM.

**Figure 4:**
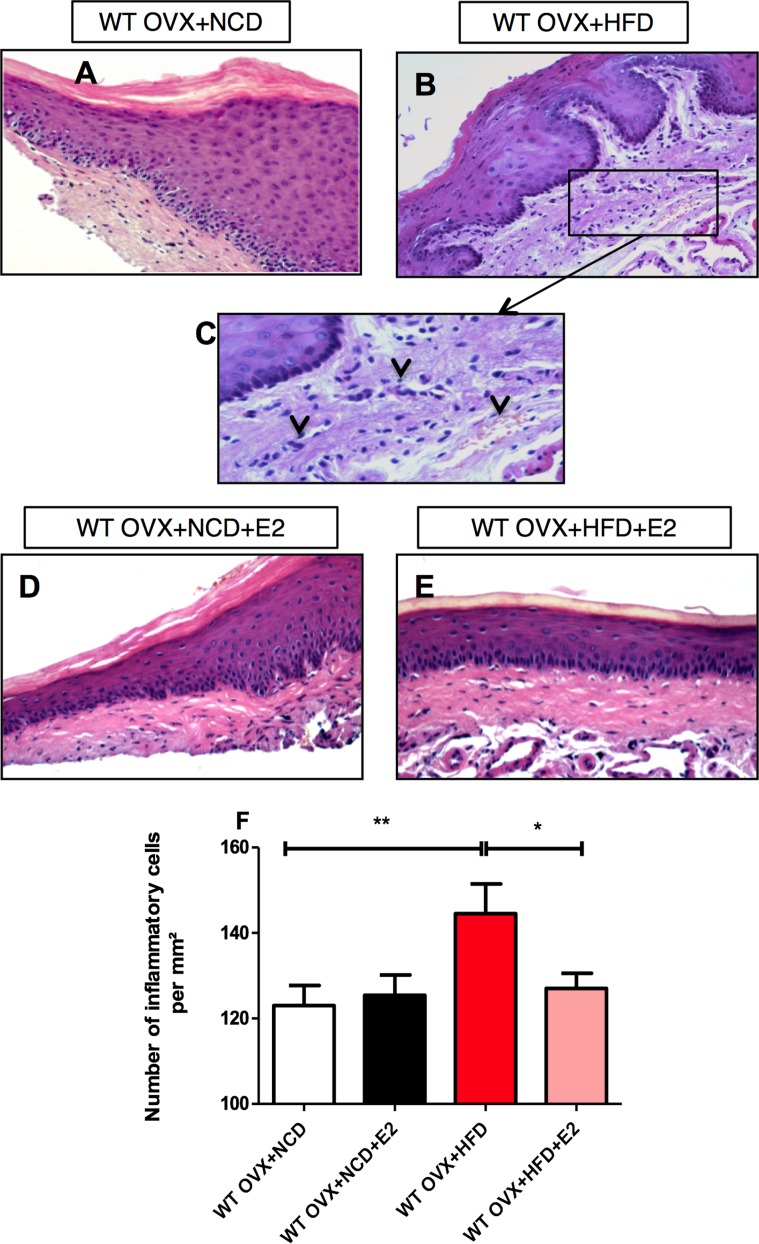
HFD-induced inflammatory cell infiltrate in gingival connective tissue is modulated by E2 in wild-type mice. Histological appearance of the gingival tissues with H/E coloration from WT OVX+NCD (**A**; n = 10),WT OVX+HFD (**B**; n = 10), WT OVX+NCD+E2 (**D**; n = 10), and WT OVX+HFD+E2 (**E**; n = 10). **C** Magnification of the inflammatory gingival tissue from HFD-fed mice. **F**. Number of inflammatory cells for each group. *P<0.05, **P<0.01, ***P<0.001 (one-way ANOVA followed by Tukey test). Results are presented as means ± SEM.

### Real-Time Quantitative PCR (qPCR) Analysis

Total RNA from white adipose tissue, liver and gingival tissue was extracted using the TriPure reagent (Roche, Basel, Switzerland). cDNA was synthesized using a reverse transcriptase (Applied Biosystems, Fost City, USA) from 1 µg of total RNA. The primers (Eurogentec, San Diego, USA) used were (5′ to 3′): tumor necrosis factor-α (*TNF-α*), forward TGGGACAGTGACCTGGACTGT; reverse TCGGAAAGCCCATTTGAGT; Interleukin 1β(*IL-1β*) forward TCGCTCAGGGTCACAAGAAA; reverse CATCAGAGGCAAGGAGGAAAAC; plasminogen activator inhibitor-1(*PAI-1*) forward ACAGCCTTTGTCATCTCAGCC; reverse CCGAACCACAAAGAGAAAGGA and interleukin-6 (*IL-6*) forward ACAAGTCGGAGGCTTAATTACACAT; reverse TTGCCATTGCACAACTCTTTTC. The concentration of each mRNA was normalized for RNA loading for each sample using ribosomal protein L19 (*RPL19*) forward GAAGGTCAAAGGGAATGTGTTCA; reverse CCTTGTCTGCCTTCAGCTTGT, as an internal standard and the data were analysed according to the 2 ^−ΔΔCT^ method [24].

**Figure 5:**
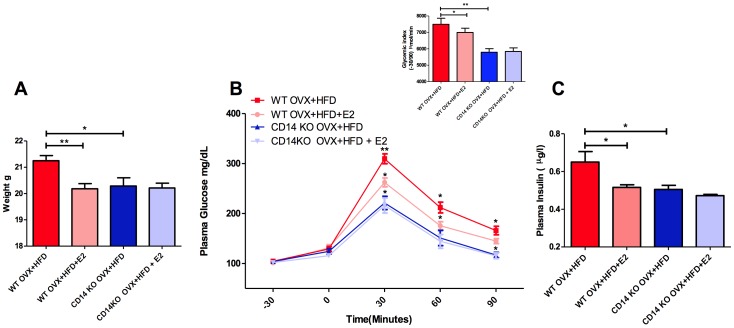
HFD-fed *CD14*KO mice did not exhibit metabolic disorders compared with WT. A) Body weight was assessed in 8-wk-old mice after 4 weeks of diet : WT OVX+HFD (n = 10), WT OVX+HFD+E2 (n = 10), CD14KO OVX+HFD (n = 12) and CD14KO OVX+HFD+E2 (n = 12). **B**) Time course of glycemia (mg/dl) during an IPGTT. The inset represents the glycemic index for each group. **C**) Fasted plasma insulin concentration (µg/l) after 4 weeks of diet : WT OVX+HFD (n = 6), WT OVX+HFD+E2 (n = 6), CD14KO OVX+HFD (n = 6) and CD14KO OVX+HFD+E2 (n = 6). *P<0.05, **P<0.01 (One-way ANOVA followed by Tukey’s post-test for **A** and **C**; and Two-Way ANOVA with Bonferroni’s post-test for **B**). Results are presented as means ± SEM.

### Culture and Identification of Periopathogens

The composition of the sub-gingival microbiota was determined as previously described [10]. The selected site was cleaned with 75% ethanol to remove the supragingival bacterial biofilm. Cervicular fluid was sampled with three endodontic sterile paper points held in sterile pliers: paper points were inserted into the sub-gingival space and then placed in a 2-ml bottle of reduced transport medium VGMA-III of Moëller. After mixing for 30 sec at maximal speed on a Vortex mixer, the 2-ml bottles containing glass beads were opened in an anaerobic chamber and samples were serially diluted ten-fold in Wilkins–Chalgren broth (WC, Oxoid, Basingstoke, Hampshire, UK). Bacteria were cultured on non-selective or selective medium agar plates [10]. Identification of putative anaerobic bacteria was carried out according to Bergey’s manual criteria [25]. Genomic bacterial DNA was extracted using a classical phenol/chloroform method followed by alcohol precipitation (ice-cold 70% alcohol vol/vol). Semi-quantitative PCR was carried out using 2 µl of the extracted DNA with specific primers [4]. The primers (Eurogentec, San Diego, USA ) used were (5′ to 3′): *Fusobacterium nucleatum* forward AAGCGCGTCTAGGTGGTTATGT and reverse TGTAGTTCCGCTTACCTCTCCAG and *Prevotella intermedia* forward TCCACCGAT GAATCTTTGGTC and reverse ATCCAACCTTCCCTCCACTC.

**Figure 6:**
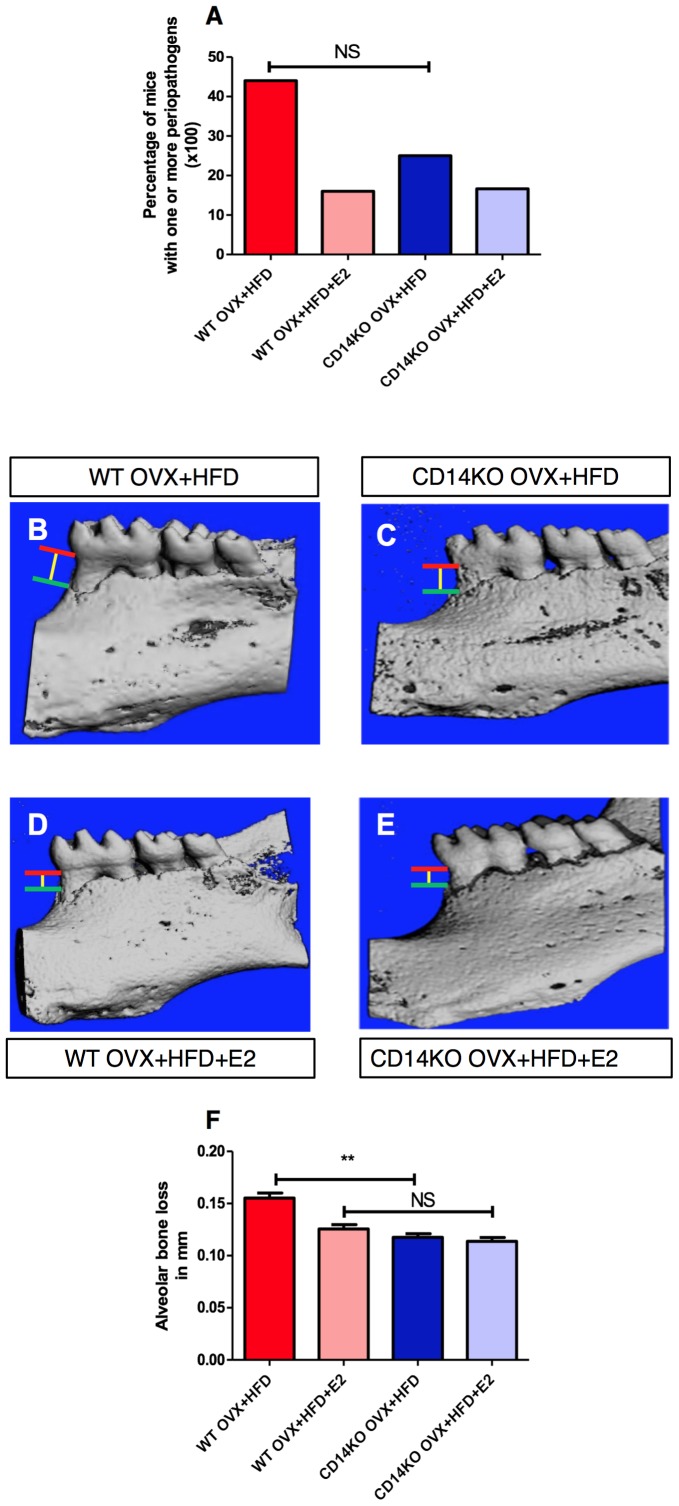
HFD-fed *CD14*KO mice are protected from periodontal disease compared with WT. A ) The occurrence of periodontal pathogens was analyzed in 8-wk-old mice after 4 weeks of diet: WT OVX+HFD (n = 10), WT OVX+HFD+E2 (n = 10), CD14KO OVX+HFD (n = 12) and CD14KO OVX+HFD+E2 (n = 12). **B–G**) Hemi-mandible from each group, as reconstructed by the micro-CT. **F** CEJ (red line: cemento-enamel junction)-ABC (green line: alveolar bone crest) distance to represent alveolar bone loss (yellow line). *P<0.05, **P<0.01, ***P<0.001 (one-way ANOVA followed by Tukey test). Results are presented as means ± SEM.

### Histological Examination

Gingival tissue surrounding the lower molars were excised, fixed in 4% paraformaldehyde for 48 hours and embedded in paraffin. Sections (4 µm thick), were then stained with hematoxylin/eosin. To quantify the infiltration of inflammatory cells, immune cells were counted in ten microscopic fields randomly selected from each group.

**Figure 7:**
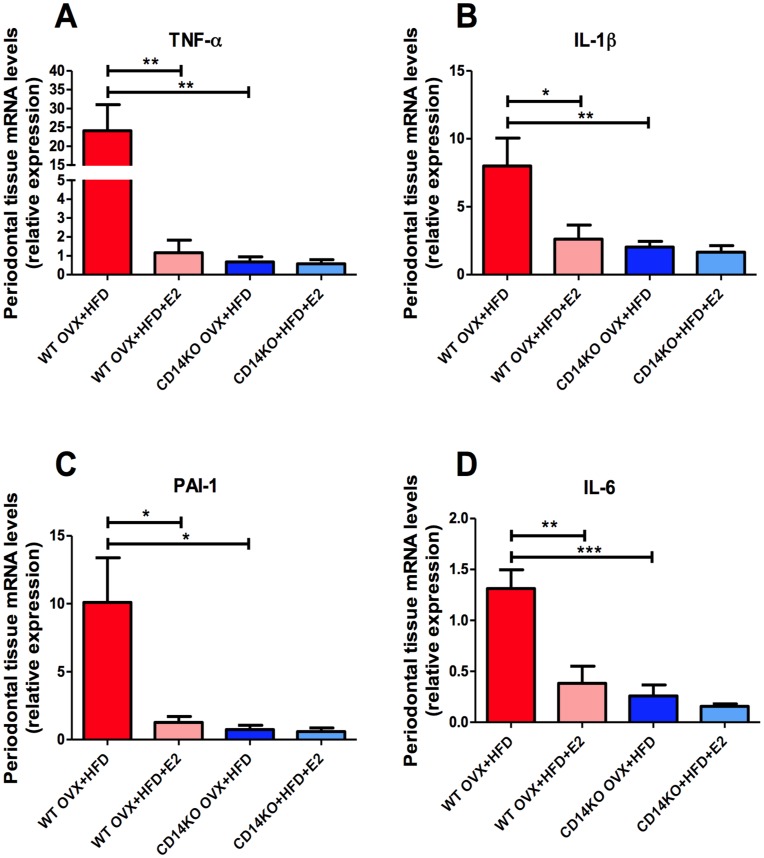
*CD14*KO mice did not display HFD-induced periodontal inflammation. mRNA expression of TNF-α (**A**), IL-1β (**B**), PAI-1(**C**) and IL-6 (**D**) in gingival tissue. *P<0.05, **P<0.01,***P<0.001 (one-way ANOVA followed by Tukey test). Results are presented as means ± SEM.

**Figure 8:**
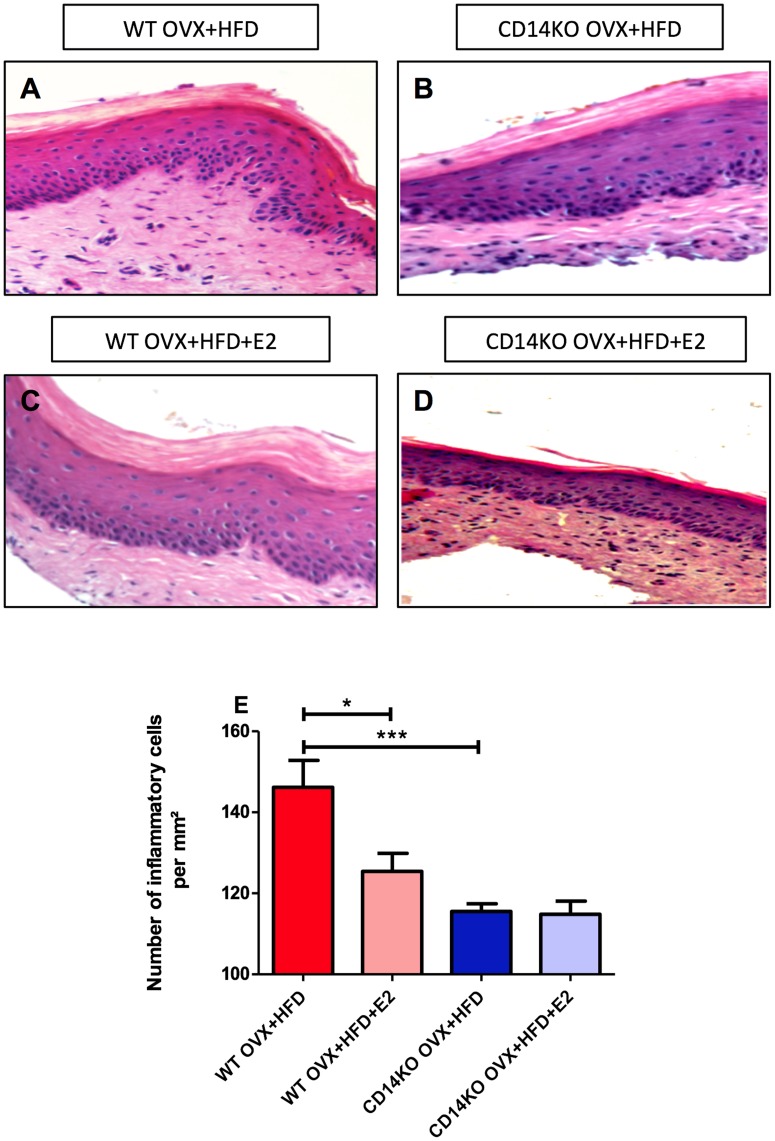
The depletion of *CD14* reduced HFD-induced inflammatory cell infiltration into gingival connective tissue. Histological appearance of the gingival tissues with H/E coloration from WT OVX+HFD (**A,** n = 6), *CD14*KO OVX+HFD (**B,** n = 6) WT OVX+HFD+E2 (**C,** n = 6), and *CD14*KO OVX+HFD+E2 (**D,** n = 6) **E**. Number of inflammatory cells for each group. *P<0.05,**P<0.01, ***P<0.001 (one-way ANOVA followed by Tukey test). Results are presented as means ± SEM.

### Quantification of Mandibular Alveolar Bone Resorption

To evaluate mandibular alveolar bone loss, hemi-mandibles were scanned using a high-resolution µCT (Viva CT40; Scanco Medical, Bassersdorf, Switzerland) [26]. Data were acquired at 45 keV, with a 10 µm isotropic voxel size. Six linear measurements were obtained from each molar by using a stereomicroscope with an on-screen computer-aided measurement package. The alveolar bone loss (in mm) was measured from the cemento-enamel junction (CEJ) to the alveolar bone crest (ABC) for each molar [27]. Three-dimensional reconstructions were generated from a set of 400 slices. After micro-CT analysis, the hemi-mandibles were cleaned with 0.1% hypochlorite over 20 min and then stained with 10% methylene blue.

### Statistical Analysis

Results are presented as means ± SEM. One-way ANOVA followed by Tukey’s post-test was used to assess the statistical significance between groups, except for the IPGTT analysis, where two-way ANOVA followed by Bonferroni’s post-test was applied. The effects of diet, estrogen supplementation and *CD14* deletion on periodontal microbiota composition were tested using Fisher’s exact test. A two-sided p-value <0.05 was considered statistically significant. Statistical analyses were performed using GraphPad Prism version 5.00 for Windows Vista (GraphPad Software, San Diego, CA), and R (version 2.12.1).

## Results

### Estradiol Prevents HFD-induced Metabolic Disorders in WT Ovariectomised Mice

Four-week-old wild-type C57Bl6/J female mice were ovariectomised then randomised according to both diet and E2-treatment, as reported in **Figure 1**. As expected, in comparison to NCD-fed mice, mice fed with a HFD for four weeks developed a dysmetabolic phenotype characterised by increased body weight gain, impaired fasting glycemia, hyperinsulinemia and glucose-intolerance. It was noteworthy that the harmful effects of HFD on body weight, fasted plasma insulin and glucose homeostasis were largely prevented by E2-supplementation **(** **Fig. 1A–C** **)**.

### HFD Promotes Periodontitis in the Absence of Estrogens

We then examined the respective influence of a HFD and estrogens on periodontal health. Without estrogen supplementation, the HFD significantly increased the percentage of animals with at least one and up to two periopathogen species, *Pi* and *Fn,* compared with control mice maintained on the NCD (**Fig. 2A** **;** **Table 1** **)**. Furthermore, the HFD instigated the presence of *Pi/Fn* association in the periodontal microbiota of OVX-mice (**Table 1** **)**, and induced alveolar bone resorption (a common feature of periodontitis) (**Fig. 2B–F**). Interestingly, E2-administration to OVX mice blunted the deleterious effects of the HFD on alveolar bone (**Fig. 2B–F** **)** and modified the periodontal microbiota by reducing the percentage of mice with *Pi* and *Fn* association (**Table 1**).

### E2-treatment Prevents HFD-induced Inflammation of Periodontal Tissues

To determine whether E2 prevents HFD-induced periodontal disease by controlling inflammatory processes, we assayed the mRNA concentrations of several inflammatory mediators in the gingiva. TNF-α, IL-1β, PAI-1 and IL-6 expression significantly increased in the gingival connective tissue of OVX mice fed a fat-enriched diet when compared with their NCD-fed counterparts. Conversely, the proinflammatory effect of the HFD was abolished by E2-supplementation (**Fig. 3A–D**).

Microscopic examination of gingival connective tissue of HFD-fed mice highlighted an inflammatory cell infiltration (**Fig. 4A–F**), and red cells extravasation, compared with the NCD-fed OVX control mice (**Fig. 4A–F**). Interestingly, E2-supplementation reversed the HFD-induced periodontal soft tissue inflammation (**Fig. 4F**).

### *CD14*KO Mice are Protected from HFD-induced Periodontitis

CD14 is an important modulator of the inflammatory responses induced by gram-negative bacteria. We therefore assessed the impact of *CD14* deletion on HFD-associated disorders including metabolic and periodontal alterations. According to our previous data [4] and in contrast to WT mice, *CD14*KO mice did not show increased body weight, fasted glycemia and plasma insulin concentration or glucose-intolerance, when fed a HFD (**Fig. 5A–C**) or a NCD (**Fig. S1A**) for four weeks.

Interestingly, the deletion of *CD14* also prevented the HFD-induced alveolar bone loss (**Fig. 6B–F**), although the association of *Pi*/*Fn* was unaffected in *CD14*KO HFD-fed mice (WT OVX+HFD 25% vs *CD14*KO OVX+HFD 24% **Table 1**). Like WT NCD-fed mice, *CD14*KO NCD-fed mice did not show a modified periodontal microbiota (**Table S1**). Furthermore, in contrast to the protective effect observed in WT mice, estrogen supplementation had no effect on the alveolar bone level in *CD14*KO mice (**Fig. 6A–F**).

The TNF-α, IL-1β, PAI-1 and IL-6 expression in the periodontal soft tissue of HFD-fed *CD14*KO mice was comparable to the controls (**Fig. 7A–D**). Moreover, in contrast to HFD-fed WT mice (**Fig. 8A–E**), the immune cell infiltration in gingival tissue was dramatically reduced in HFD-fed *CD14* mutant mice. In addition, E2 did not affect alveolar bone loss and overall inflammatory tone in *CD14*KO OVX+NCD (**Fig. S2A–F**).

## Discussion

The data presented in this study showed that a fat-enriched diet combined with estrogen deficiency induced a periodontal disease in mice. Furthermore, our data strongly suggest that estradiol replacement may prevent HFD-induced metabolic and periodontal disorders.

Medium- or long-term diabetic patients with degenerative multi-organ complications [28], are also prone to frequent and severe periodontitis. To date, the mechanisms linking diabetes to periodontal diseases remain poorly understood. Our data showed that HFD increases the proportion of mice harbouring periodontal pathogens such as *Fusobacterium nucleatum* and *Prevotella intermedia*, known to be part of the subgingival plaque in periodontal pockets [29], [30]. Indeed, it has been suggested that a fat-enriched diet may enhance the occurrence of oral pathogens in diabetic patients [31]. The reasons for this fundamental modification are still unclear. However, a switch to a high-fat diet induces a new intestinal ecology and, hence, a new periodontal ecology [32]. This modification increased the proportion of gram-negative bacteria producing inflammatory LPS, at least in the intestine [4]. Furthermore, the microbial diversity could explain the different metabolic phenotypes [33]. Therefore, an inappropriate immune function in reaction to the many bacterial antigens would generate a metabolic inflammation and the corresponding immune messengers may impair and maintain the metabolic disorders [2]. On the other hand, a microbiota composed of gram-negative bacteria produces LPS that directly move into the blood in human [34] or animal models to aggravate inflammation and systemic diseases [12], [35]. Indeed, HFD-induced metabolic endotoxemia (an increase in plasma levels of LPS) was shown to be an initiator of metabolic diseases [35] *via* enhanced systemic inflammation [2]. Hence, the elevated systemic inflammation in diabetic patients could be linked to PD [36], demonstrating a two-sided relationship between these pathologies. Thus, inflammation could be a key feature of periodontitis to be targeted [5] and we can postulate that a fat-enriched diet may be a regulator of the relationship between microbiota and human host [36].

Although the mechanism through which periopathogens contribute to periodontal diseases is still not fully understood, studies have suggested that the pathogenic microbiota induces local and systemic inflammation [37], [38], a common feature of metabolic disease [2]. As reported above, periodontitis is characterized by a complex biofilm composed of LPS-harboring gram-negative bacteria [32]. CD14, a cell-surface molecule involved in innate immunity [39], is a systemic modulator of LPS-induced metabolic disorders. In this context, we have demonstrated in the current study that *CD14-*ablation protects against HFD-induced inflammation-triggered diabetes and periodontitis. Indeed, HFD-fed *CD14*KO ovariectomised mice did not show any features of periodontitis such as alveolar bone loss or immune cell infiltration into the gingival connective tissue. Nevertheless, the number of mice exhibiting a periopathogenic microbiota was found to be increased. This result may suggest that a HFD induces metabolic disorders and periodontal damage through activation of innate immunity via LPS production by periodontal microbiota, rather than direct adverse effects on tissues by periopathogenic derivatives as previously suggested [40]. In agreement with our findings, previous studies have reported relationships between CD14, inflammation and PD in humans [41]. Therefore, the use of antibiotic treatment restricted to the periodontal pocket could be thought.

Homeostasis in periodontium involves multiple factors including variation in sex hormone production [42]. Many studies recently showed that modification of estrogenic status during menstrual cycles [43], puberty [44] and pregnancy [42], could be linked to the occurrence of gingivitis, an inflammation of soft periodontal tissue without loss of attachment [42]. Some studies hypothesized that estrogen therapy may be used in these periodontal pathologies [45]. However, this therapeutic strategy would be not applicable in gingivitis because it is spontaneously reversed after gingival plaque removing and/or menstrual cycle normalization [46]. Conversely, estrogen deficiency is considered a risk factor for periodontitis [19]. Importantly, we recently demonstrated that estrogen replacement reversed HFD-induced metabolic diseases [18], [20]. Here, we report the reversal of the proportion of WT OVX mice with periodontal pathogenic microbiota by E2-treatment, as already reported in post-menopausal women using hormone replacement therapy [23]. While *in vivo* E2-administration enhanced systemic inflammation in mice [47], our results suggest that estrogen supplementation may have a specific protective effect on periodontal tissue by regulating the inflammation induced by a HFD [48]. Since E2 increases the thickness and keratinization of epithelia [49] we suggest that this hormone could reinforce the gingival epithelium against the colonization by specific periopathogens [48]. Moreover, E2 stimulates the innate immune response [50], [51], increasing macrophage reactivity against aggressive bacteria [52]. As already suggested [53], our results support the hypothesis that E2-supplementation may have anti-inflammatory effects on periodontal tissue. Thus, we can propose that our present results could be the basis of further experimental trials dedicated to show the importance of hormone replacement therapy as well as that of a tight glycemic control to maintain periodontal health.

In conclusion, the data reported herein suggest a causal link between the activation of the LPS pathway on innate immunity by periodontal microbiota and the occurrence of HFD-induced periodontal defects. This pathophysiological mechanism could be targeted by estrogens, which may thus represent a new therapeutic perspective to prevent HFD-induced periodontal inflammation and reduce the occurrence of PD.

## Supporting Information

Figure S1 **NCD-fed CD14KO mice did not exhibit metabolic disorders. A)** Body weight was assessed in 8-wk-old mice after 4 weeks of diet: CD14KO OVX+NCD (n = 12) and CD14KO OVX+NCD+E2 (n = 12). **B)** Time course of glycemia (mg/dl) during IPGTT. The inset represents the Glycemic index for each group. *P<0,05 (one-way ANOVA followed by Tukey test for A and Two-Way ANOVA with Bonferroni’s post-test for B). Results are presented as means ± SEM.
(TIF)

Figure S2 **NCD-fed CD14KO mice did not display periodontal disease.** **A)** Alveolar bone loss of Hemi-mandibule from each group : CD14KO OVX+NCD (n = 5) and CD14KO OVX+NCD+E2 (n = 5), was explored B.C.D.E mRNA expression of TNF-α **(B)**, IL-1β **(C),** PAI-1**(D)** and IL-6 **(E)** in gingival tissue. D. Number of inflammatory cells for each group *P<0,05 **P<0,01 ***P<0,001 (one-way ANOVA followed by Tukey test). Results are presented as means ± SEM.
(TIF)

Table S1 **Rates of positive bacterial cultures in NCD-fed** **mice.** The table shows the qualitative analysis of periodontal microbiota in each group : WT OVX NCD E2 (n = 16), CD14KO OVX+NCD (n = 5) and CD14KO OVX+NCD+E2 (n = 5). *P<0.05,**P<0.01 (Fischer exact tests).
(PDF)
