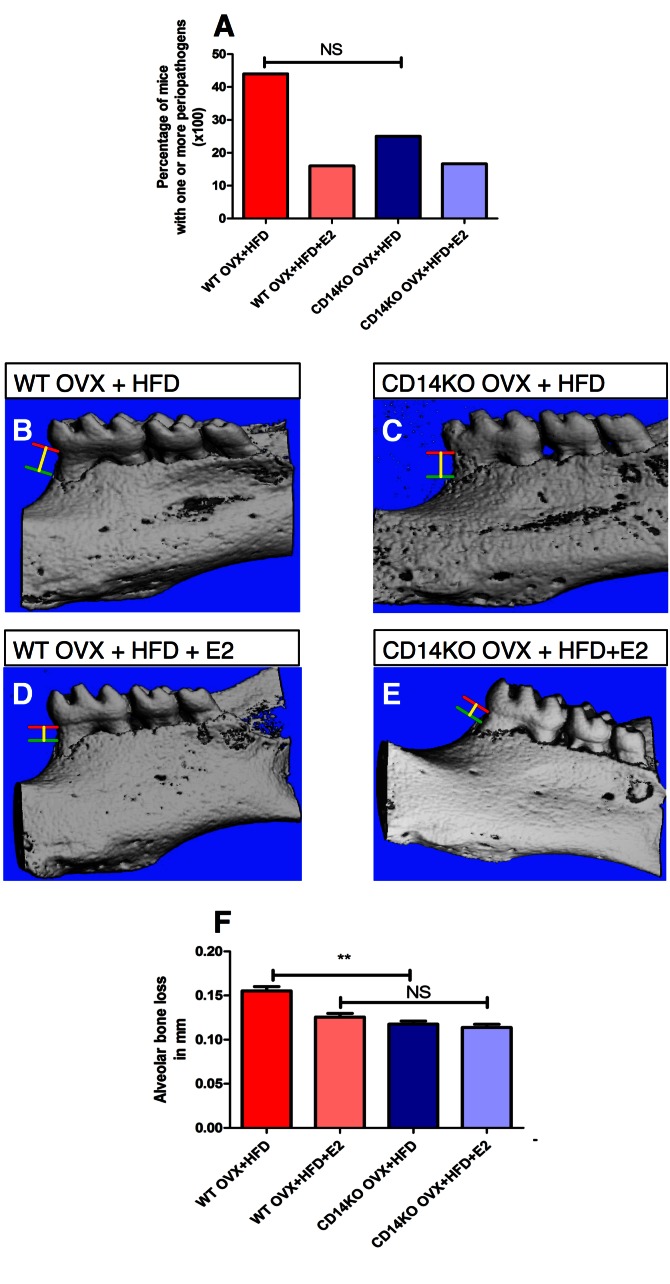# Correction: High-Fat Diet Induces Periodontitis in Mice through Lipopolysaccharides (LPS) Receptor Signaling: Protective Action of Estrogens

**DOI:** 10.1371/annotation/83ddaae2-ce5d-4683-bbff-f87945a2fa2c

**Published:** 2013-05-20

**Authors:** Vincent Blasco-Baque, Matteo Serino, Jean-Noël Vergnes, Elodie Riant, Pascale Loubieres, Jean-François Arnal, Pierre Gourdy, Michel Sixou, Rémy Burcelin, Philippe Kemoun

Figure 6 is incorrect. The correct Figure 6 can be viewed here: 

**Figure:**