# Supplementary figures and images for: High-Fat Diet Induces Periodontitis in Mice through Lipopolysaccharides (LPS) Receptor Signaling: Protective Action of Estrogens
Source: PLoS One. 2012 Nov 2;7(11):e48220. doi: 10.1371/journal.pone.0048220 (PMC3487901; doi:10.1371/journal.pone.0048220)

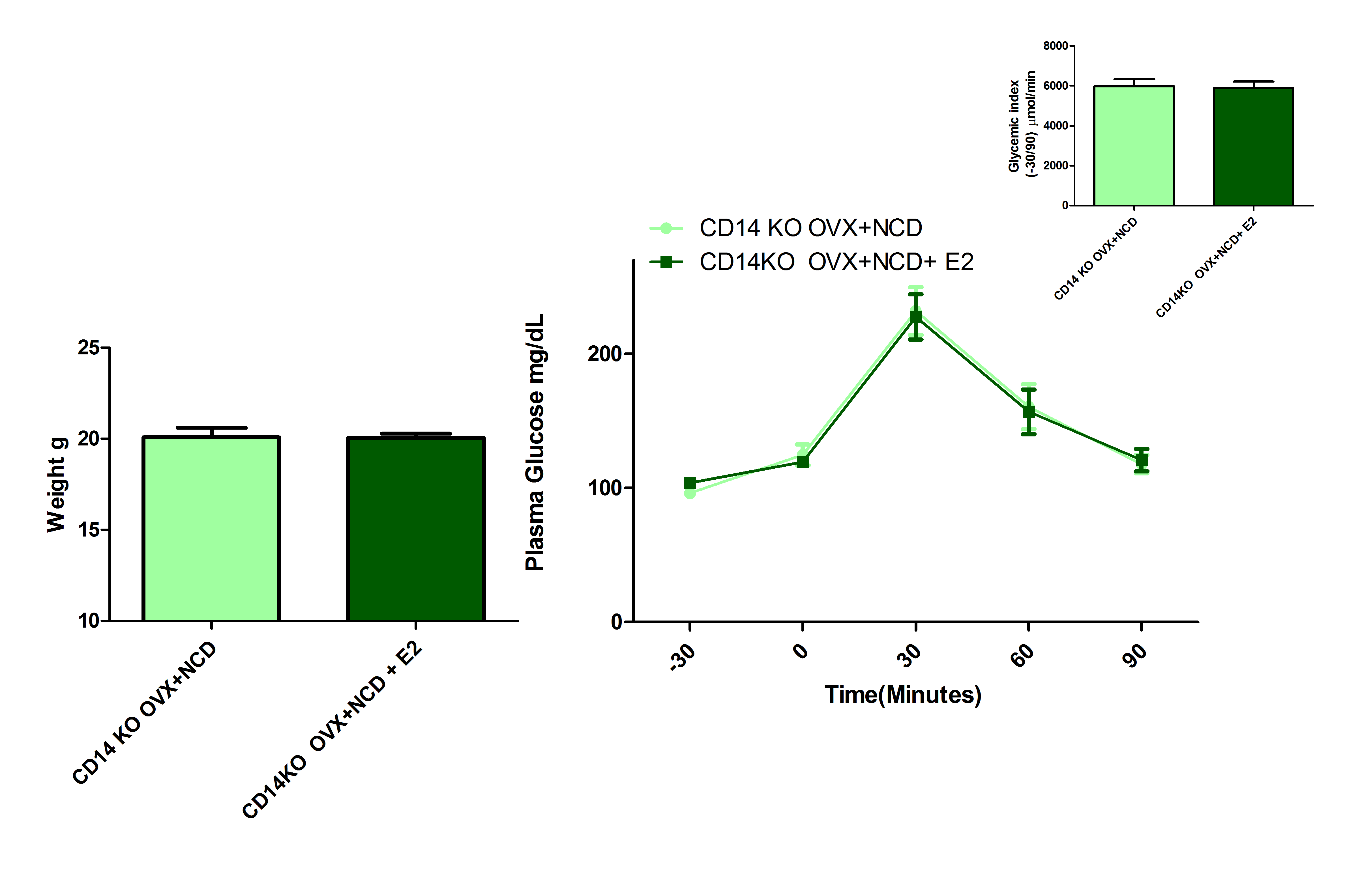

Supplement: Figure S1 — NCD-fed CD14KO mice did not exhibit metabolic disorders. A) Body weight was assessed in 8-wk-old mice after 4 weeks of diet: CD14KO OVX+NCD (n = 12) and CD14KO OVX+NCD+E2 (n = 12). B) Time course of glycemia (mg/dl) during IPGTT. The inset represents the Glycemic index for each group. *P<0,05 (one-way ANOVA followed by Tukey test for A and Two-Way ANOVA with Bonferroni’s post-test for B). Results are presented as means ± SEM. (TIF) [file pone.0048220.s001.tif]

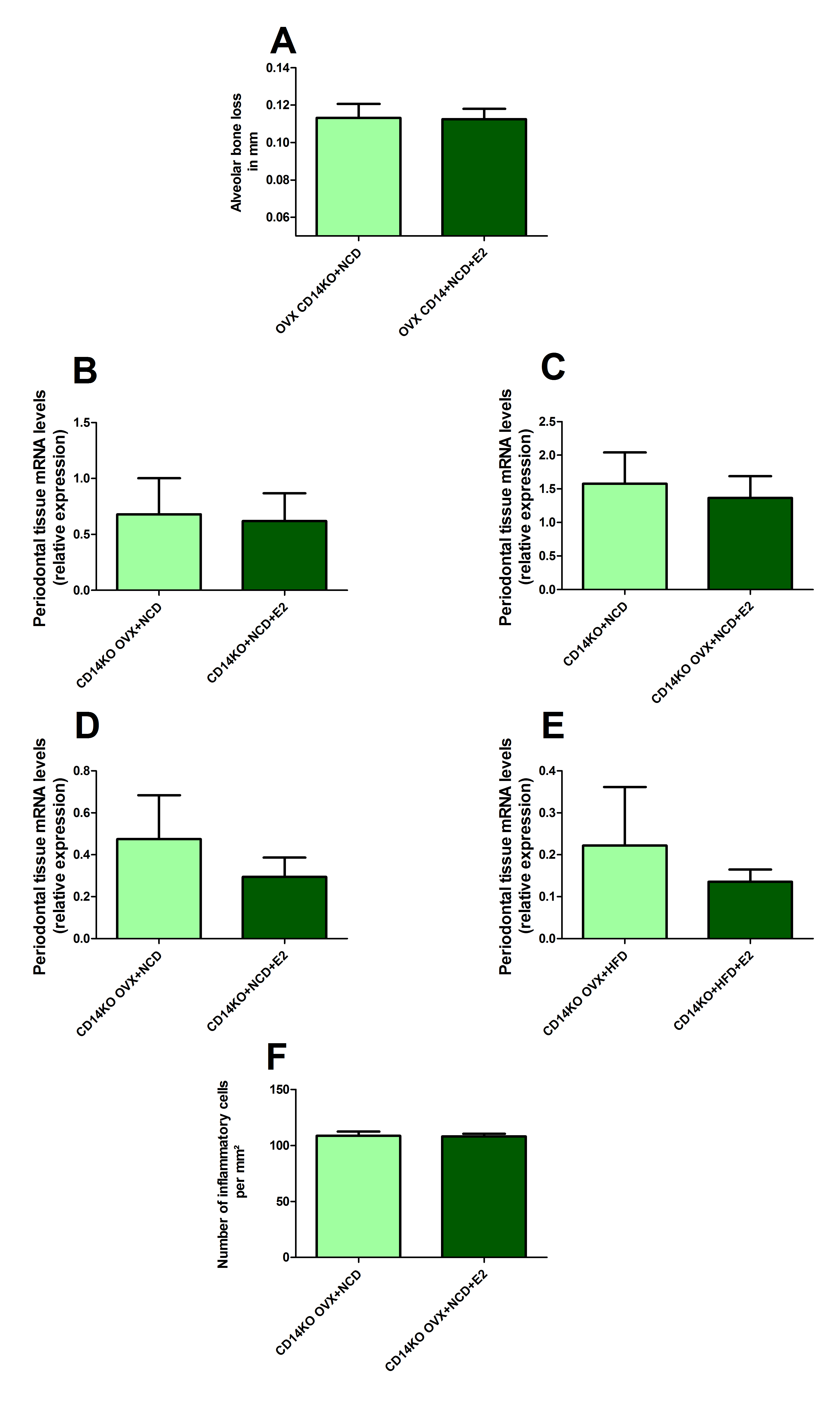

Supplement: Figure S2 — NCD-fed CD14KO mice did not display periodontal disease. A) Alveolar bone loss of Hemi-mandibule from each group : CD14KO OVX+NCD (n = 5) and CD14KO OVX+NCD+E2 (n = 5), was explored B.C.D.E mRNA expression of TNF-α (B), IL-1β (C), PAI-1(D) and IL-6 (E) in gingival tissue. D. Number of inflammatory cells for each group *P<0,05 **P<0,01 ***P<0,001 (one-way ANOVA followed by Tukey test). Results are presented as means ± SEM. (TIF) [file pone.0048220.s002.tif]
