# Supplementary material for: High-Fat Diet Induces Periodontitis in Mice through Lipopolysaccharides (LPS) Receptor Signaling: Protective Action of Estrogens
Source: PLoS One. 2012 Nov 2;7(11):e48220. doi: 10.1371/journal.pone.0048220 (PMC3487901; doi:10.1371/journal.pone.0048220)
Supplement: Table S1 — Rates of positive bacterial cultures in NCD-fed mice. The table shows the qualitative analysis of periodontal microbiota in each group : WT OVX NCD E2 (n = 16), CD14KO OVX+NCD (n = 5) and CD14KO OVX+NCD+E2 (n = 5). *P<0.05,**P<0.01 (Fischer exact tests). (PDF) [file pone.0048220.s003.pdf]

**Bacterial cultures**			
	**WT OVX NCD E2**<br><br>n=16 (%)	***CD14*KO OVX NCD**<br><br>n= 12 (%)	***CD14*KO OVX NCD E2**<br><br>n=12 (%)
***Fusobacterium nucleatum* (Fn)**	0 (0)	0 (0)	1 (8.33)
***Prevotella intermedia* (Pi)**	1 (6.25)	0 (0)	0 (0)
**Both Fn + Pi**	0 (0)	1 (8.33)	0 (0)
**Fn and/or Pi**	1 (6.25)	1 (8.33)	1 (8.33)
